# Role of Protein Biomarkers in the Detection of High-Grade Disease in Cervical Cancer Screening Programs

**DOI:** 10.1155/2012/289315

**Published:** 2012-02-28

**Authors:** Charlotte A. Brown, Johnannes Bogers, Shaira Sahebali, Christophe E. Depuydt, Frans De Prins, Douglas P. Malinowski

**Affiliations:** ^1^BD Diagnostics, Women's Health and Cancer, Durham, North Carolina, USA; ^2^Laboratory for Clinical and Molecular Pathology (RIATOL), Sonic Healthcare Benelux, Antwerp, Belgium; ^3^AMBIOR, Laboratory of Cell Biology and Histology, Faculty of Medicine, University of Antwerp, Antwerp, Belgium; ^4^AZ Jan Palfijn, Antwerp, Belgium

## Abstract

Since the Pap test was introduced in the 1940s, there has been an approximately 70% reduction in the incidence of squamous cell cervical cancers in many developed countries by the application of organized and opportunistic screening programs. The efficacy of the Pap test, however, is hampered by high interobserver variability and high false-negative and false-positive rates. The use of biomarkers has demonstrated the ability to overcome these issues, leading to improved positive predictive value of cervical screening results. In addition, the introduction of HPV primary screening programs will necessitate the use of a follow-up test with high specificity to triage the high number of HPV-positive tests. This paper will focus on protein biomarkers currently available for use in cervical cancer screening, which appear to improve the detection of women at greatest risk for developing cervical cancer, including Ki-67, p16^INK4a^, BD ProEx C, and Cytoactiv HPV L1.

## 1. Introduction

Cervical cancer is the second most common cancer in women worldwide and remains a major cause of morbidity and mortality. Since the Pap test was introduced in the 1940s, there has been an approximately 70% reduction in the incidence of squamous cell cervical cancers in many developed countries by the application of organized and opportunistic screening programs. The efficacy of the Pap test, however, is hampered by high interobserver variability and high false-negative and false-positive rates [1–3]. However, as cervical cancer evolves through well-defined noninvasive intraepithelial stages, which can be distinguished morphologically, repeated screening at frequent intervals can maintain high levels of protection.

Investigators have attempted by various means to enhance the sensitivity of the Pap test. First by the introduction of liquid-based methods to address issues of specimen collection and preparation, and later by the use of computer-assisted screening systems to address screening errors and to improve screening efficiency and disease detection. Testing for oncogenic, high-risk human papillomavirus (hrHPV) DNA has been accepted as an adjunct to borderline/ASC-US cytology in primary screening. The utility of HPV as a reflex test within the ASC-US patient population is largely reflected in its negative predictive value (NPV), where an HPV negative result indicates a low likelihood of finding CIN2+ lesions upon colposcopy [4]. This triage application, however, has a very low specificity and correspondingly low positive predictive value for finding CIN2+ disease. Another testing algorithm involving cytology and HPV cotesting was reported to lead to earlier detection of CIN3+ lesions, supporting a lengthening of the screening interval [5]. In this cotesting algorithm, the combined use of cytology plus HPV testing has a very high NPV. As such, it is useful to screen a presumed normal and healthy population for the presence of CIN2+ disease and to stratify patients into two groups: (i) HPV negative, cytology normal patients who are at low risk for developing CIN2+ disease and thus qualify for an extended screening interval and (ii) patients who are either cytology abnormal or HPV positive and who are at increased risk for developing CIN2+ and require more active surveillance. Most recently, the use of hrHPV DNA detection as a primary screening test for cervical disease has been investigated [6–10]. The majority of hrHPV infections, however, induce low-grade precursor lesions, which are cleared spontaneously within one to two years of exposure, with less than 10% eventually progressing to high-grade lesions or invasive cancer. Thus, while hrHPV DNA testing has a very high sensitivity for the detection of high-grade cervical disease, it has a very low specificity and positive predictive value, thus would always require a follow-up test prior to treatment to avoid unnecessarily raising patient anxiety levels and referrals to invasive colposcopic procedures.

**Figure 1:**
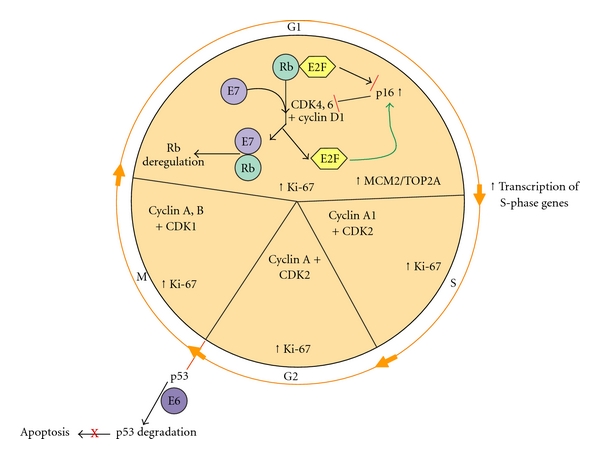
Cell cycle alterations induced by HPV E6 and E7 oncogenes in cervical neoplasia, adapted from Malinowski [11]. The presence of E7 disrupts G1-S phase regulation through the interference with E2F-Rb binding. P16INK40 is strongly expressed due to the loss of Rb/E2F repression and the strong activation by free E2F. The release of E2F results in the increased transcription of S-phase genes, including MCM2 and TOP2A. The interaction of E6 with p53 results in p53 ubiquitination and subsequent degradation, resulting in the abrogation of apoptosis. The proliferation marker Ki-67 is increased in the presence of cell cycle dysregulation caused by the E6 and E7 oncogenes.

The use of biomarkers in both cervical cytology and histology has demonstrated the ability to overcome issues with both false-positive and false-negative results, leading to improved positive predictive value of cervical screening results. Numerous protein biomarkers for the detection of cervical disease have been identified. Many of these proteins are involved in cell cycle regulation, signal transduction, DNA replication, and cellular proliferation (reviewed in [11–13]). The altered expression of these proteins is a consequence of the binding of the high-risk HPV E6 and E7 oncogenes to host regulatory proteins, resulting in the degradation of the p53 tumor suppressor gene product and the inactivation of the retinoblastoma protein leading to dysregulation of the cell cycle (Figure 1).

In contrast to the cellular markers, the HPV L1 capsid protein is a virus-specific protein and a major stimulus of the immune system used within the HPV vaccines.

This paper will focus on protein biomarkers currently under investigation for use in cervical cancer screening that appear to improve the detection of women at greatest risk for developing cervical cancer, including Ki-67, p16^INK4a^, BD ProEx C, and Cytoactiv HPV L1. These biomarkers are reported to have a role in the triage of indeterminate cytology cases, discrimination of true high-grade cervical dysplasia from mimics in histology, and may serve as predictive markers to identify lesions most likely to progress to high-grade cervical disease and cancer. The staining characteristics and reported usage of the various biomarkers in cervical cancer screening are summarized in Table 1.

## 2. Biomarkers Used in Cervical Screening

### 2.1. Ki-67

Ki-67 is a nuclear and nucleolar protein expressed during the G1, S, G2, and M phase of the cell cycle, while not being present in resting cells (G0 phase), and can, therefore, provide an index of the cell growth fraction. While the function of the Ki-67 protein remains unclear, its expression appears to be an absolute requirement for progression through the cell-division cycle [14, 15]. Since HPV infection leads to increased epithelial cell proliferation in infected tissues, increased Ki-67 staining can be an indicator of HPV infection. In normal human cervical squamous mucosa, expression of Ki-67 is limited to the proliferating basal and parabasal cells. In dysplasia and carcinoma, however, expression extends above the basal one third of the epithelium and the number of positive cells increase, with a significant positive correlation between ascending grade of squamous intraepithelial lesion and labeling index (Figure 3) [16]. The most commonly used antibody for immunohistochemical detection of the Ki-67 antigen is clone MIB-1. 

### 2.2. p16^INK4a^

The protein p16^INK4a^ is a cell-cycle regulator, with its expression tightly controlled in normal cells. This tumor suppressor protein inhibits cycle-dependant kinases 4 and 6, which phosphorylate the retinoblastoma (Rb) protein [17, 18]. Usually, binding of Rb to E2F blocks E2F-driven cell cycle activation and entry into the S-phase of the cell cycle. In a transforming HPV infection, however, the viral oncogene E7 disrupts the binding of the Rb protein to the E2F transcription factor, resulting in drastically increased levels of p16^INK4a^ [19, 20], the detection of which can serve as a surrogate biomarker for persistent infection with high-risk HPV (Figure 1). It is widely accepted that p16^INK4a^ is a sensitive and specific marker of dysplastic cells of the cervix and is a useful biomarker in cervical cancer lesion diagnosis and cervical screening [21–25]. Multiple antibodies to p16 have been utilized in research studies; however, the E6H4 clone (CINtec, mtm laboratories AG, Heidelberg, Germany) appears to be most commonly used. A dual p16/Ki-67 immunocytochemistry assay is now also available for use as an adjunctive test in cervical cancer screening (CINtec Plus, mtm laboratories AG, Heidelberg, Germany).

### 2.3. BD ProEx C

BD ProEx C is a protein-based biomarker reagent (BD Diagnostics, Burlington, NC, USA) containing antibodies to the nuclear proteins minichromosome maintenance protein 2 (MCM 2) and topoisomerase II alpha (TOP2A), proteins that have been shown to accumulate in HPV-transformed cells. BD ProEx C staining is limited to the basal proliferating layer of normal cervical epithelium and is absent in differentiated and quiescent cells. In contrast, in cervical glandular and squamous dysplasia, BD ProEx C expression is dramatically increased, due to the increased transcription of S-phase genes (aberrant S-phase induction) resulting from the action of the oncogenic HPV E7 protein.

The minichromosome maintenance (MCM) proteins function in the early stages of DNA replication through loading of the prereplication complex onto DNA and functioning as a helicase to help unwind the duplex DNA during de novo synthesis of the duplicate DNA strand [26, 27]. Origin licensing, which occurs before S phase in late mitosis and early G1, involves the stable loading of the minichromosome maintenance (MCM) complex comprising six replication proteins—MCM2, MCM3, MCM4, MCM5, MCM6, and MCM7 (termed MCM2-7)—onto DNA at replication origins. Expression of all six MCMs is seen throughout all phases of the cell cycle and is downregulated following exit from the cell cycle into quiescence, differentiation, or senescence, thus they are a unique marker of cells with proliferative capacity. Deregulation of MCM2-7 appears to be an early event in multistep tumorigenesis, and many studies have now shown that there is inappropriate expression of MCM2-7 in a wide variety of premalignant dysplasias and cancers [28–31].

Similar to the MCM2-7 markers, topoisomerase II-*α* (TOP2A) has been shown to be overexpressed in cervical neoplasia at both the mRNA and protein levels [32–35]. TOP2A is a nuclear enzyme that is responsible for relaxing supercoiled DNA during DNA replication and during chromosome condensation and mitosis and is required for the segregation of daughter chromosomes at the end of replication. Evaluation of TOP2A expression has shown that TOP2A overexpression is associated with the progression from CIN2 to advanced cervical neoplasia [32].

**Figure 2:**
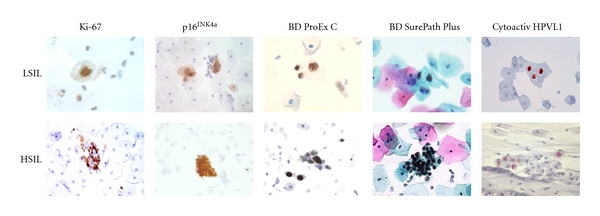
Biomarker expression in low-grade squamous intraepithelial lesions and high-grade squamous intraepithelial lesions in cervical cytology specimens. Ki-67, p16^INK4a^, BD ProEx C, and BD SurePath Plus expression detected in liquid-based cytology samples and Cytoactiv HPV L1 staining performed on conventional Pap smears (L1 images courtesy of Dr. Ralf Hilfrich).

The BD ProEx C test was designed as a reflex test to identify CIN2+ disease in women with ASC-US and LSIL cytology results. The next generation test designed for primary screening applications, BD SurePath Plus, combines on one slide traditional Pap staining for cellular morphology, in combination with immunocytochemical detection of the overexpression of two biomarkers: MCM2 and MCM7 (Figure 2).

### 2.4. Cytoactiv HPV L1 Capsid Protein

The Cytoactiv Screening antibody detects the L1 capsid protein of all known HPV types. Together with the L2 protein, the L1 capsid protein forms a protective cover for the viral genetic material. In addition, it is a ligand for a surface receptor of the host cell in the basal/parabasal cell layer of the epithelium, gaining access to the basal epithelial layer as a result of epithelial erosions or mucosal ulcerations in the transformation zone susceptible to inflammation at the cervical/endocervical junction. HPV L1 expression is found in the early, productive phase of HPV infection but is progressively lost during cervical carcinogenesis. This loss of L1 expression may result from the integration of viral DNA into the human genome, which may disrupt the L1 gene or cause loss of L1 expression by segregating the viral promoter from the L1 gene, or may reflect an abnormality in transcription factor pathways or in control of L1 protein translation [36, 37].

As the L1 capsid protein is one of main targets for T cell-mediated immune response, cells with a lack of L1 protein synthesis may escape immune system recognition, allowing disease progression. Promising data using the Cytoactiv test (Cytoimmun, Pirmasens, Germany) supports this notion, with progressive disease more frequently detected in L1-negative intraepithelial lesions [38–41].

## 3. Role of Biomarkers to Improve Cervical Cancer Screening

The use of biomarkers such as Ki-67, p16^INK4a^, and BD ProEx C has been reported to facilitate the detection of abnormal cells within a Pap cytology sample based upon simple immunocytochemistry assay formats (Figure 2). The published reports on the use of these biomarkers in Pap cytology samples have indicated their ability to triage mildly abnormal and indeterminate cytology cases, with those found to have increased levels of biomarkers staining more likely to represent cases with true high-grade cervical disease. In addition, the biomarkers can be utilized to highlight potentially abnormal cells on a background of normal, reactive or other nonmalignant cells, through colorimetric staining, directing the attention of slide screeners to cells of interest.

The utility of Ki-67 immunocytochemistry has both been shown in conventional Pap smears [42–44] and liquid-based cervical cytology [45, 46]. In patients with ASC-US and LSIL, Ki-67 immunocytochemistry demonstrated 96% sensitivity, 67% specificity, 49% PPV, and 98% NPV for detection of high-grade CIN [42]. Sahebali et al. [45] reported that receiver operating characteristic curves indicated a test accuracy (AUC) of 0.68, 0.72, and 0.86 for ASC-US, LSIL, and HSIL, respectively.

A recent meta-analysis analyzed 27 studies evaluating the use of p16^INK4a^ immunostaining in cytological specimens from the uterine cervix [24]. The proportion of cervical smears overexpressing p16^INK4a^ increased with the severity of cytological abnormality, with 12% of normal smears positive for the biomarker compared to 45% of ASC-US and LSIL, and 89% of HSIL smears. In order to improve the specificity of p16 cytology, an interpretation algorithm was developed which incorporates components of both staining and nuclear score in order to facilitate the assessment of the biomarker [47]. In an ASC-US/LSIL triage study, this scoring system resulted in 95% sensitivity and 84% specificity for ASC-US and 100% sensitivity and 82% specificity in LSIL for the detection of biopsy-proven high-grade CIN [48].

In a subset of patients from the Technologies for Cervical Cancer screening (NTCC) randomized controlled trial in Italy, the performance of p16 triage of HPV primary screen-positive women was examined [49]. Sensitivity and specificity for CIN2+ and CIN3+ of p16 immunostaining was 88% (95% CI 80–94) and 61% (633/1045; 57–64), respectively, with CIN2+ as the endpoint, and 61% (95% CI 77–97) and 59% (95% CI 55–63) for the CIN3+ endpoint, respectively. This screening algorithm was reported to produce a significant increase in sensitivity compared with conventional cytology, with no substantial increase in referral to colposcopy [39].

 The CINTec Plus biomarker cocktail, composed of antibodies against p16^INK4a^ and Ki-67, has been evaluated as a reflex test from borderline cytology results and in cytology negative, high-risk HPV positive cases [50–52]. In the European Equivocal or Mildly Abnormal Pap Cytology Study (EEMAPS), the sensitivity of the dual stain cytology for biopsy-confirmed CIN2+ was 92.2% for ASC-US cases and 94.2% for LSIL, with specificity of 80.6% and 68.0%, respectively [50]. As a triage for HPV-positive, cytology-negative cases in women ≥ 30, Petry et. al. [52] observed a 91.9% sensitivity and 82.1% specificity for CIN2+ on biopsy.

The performance of BD ProEx C in the detection of CIN2+ disease in ASC-US+ BD SurePath liquid-based cytology specimens has been reported in five studies, with sensitivity, specificity, PPV, and NPV for detection of CIN2+ disease ranging from 56.5–98%, 72-97.6%, 27.4–97%, and 95–99.3%, respectively [53–57]. A few studies have directly compared the performance of p16^INK4a^ [21, 23, 58] and BD ProEx C [55, 56] immunocytochemistry to HPV DNA detection by Hybrid Capture 2 (Qiagen, Venlo, The Netherlands) for the detection of CIN2+ disease. The PPV of both biomarkers was found to be superior to HC2 in these studies, which analyzed ASC-US, LSIL, and ASC-H liquid-based cytology specimens. Depuydt et al. [57] evaluated the efficacy of eight cervical cancer screening strategies relative to cytology with emphasis on the use of the BD ProEx C biomarker as a tool of triage following primary cytology or hrHPV testing. In the context of a reflex application from a high-risk HPV primary screen to identify CIN2+ disease, BD ProEx C was found to increase the specificity (98.3% versus 85.0%) and PPV (41.7% versus 9.3%) of screening compared to hrHPV alone, resulting in an 82% decrease in colposcopy procedures.

## 4. Improvement in the Histological Classification of Cervical Biopsies

While the cytology-based Pap test is used as the screening test for cervical cancer, histopathological evaluation of a cervical biopsy from a woman with an abnormal Pap test is the “gold standard” for the diagnosis of cervical neoplasia. However, diagnosis variability has been documented among observers and depends, in part, on the grade of the abnormality [59]. Previous studies have demonstrated that the histologic detection and grading of HPV-induced CIN, especially the low-grade categories such as atypical squamous metaplasia, HPV koilocytosis, and CIN1, have poor reproducibility and are limited by interobserver variability [60, 61]. As reactive/reparative epithelial changes, immature squamous metaplasia and atrophy are well-recognized mimics of high-grade disease and frequently cause problems in histological interpretation, there is a need in pathology practice for a biomarker reagent that will help in distinguishing true dysplasia from dysplasia mimics. The ability to make this distinction will ensure that invasive procedures, such as LEEP and cone biopsy, which can result in pregnancy complications in future pregnancies, are only performed on women with true high-grade cervical disease.

**Figure 3:**
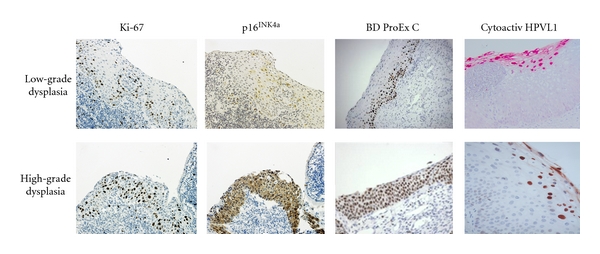
Biomarker expression in cervical intraepithelial neoplasia. The proliferative compartment progressively expands with histological grade, and this is paralleled by the appearance of immunostaining for Ki-67, p16^INK4a^, and BD ProEx C in more superficial epithelial layers, with the majority of the epithelium staining in high-grade squamous intraepithelial lesions. For Cytoactiv HPV L1, higher expression is found in low-grade lesions, with a loss of L1 protein often observed in high-grade lesions (L1 images courtesy of Dr. Ralf Hilfrich).

It has been shown that Ki-67, p16^INK4a^, and BD ProEx C immunostains are helpful in the assessment of cervical biopsies by decreasing interreader variability, assisting in the discrimination of true dysplasia from mimics, and identifying regions of focal disease that can be missed with H & E staining alone. Representative staining patterns of the biomarkers in cervical dysplasia are shown in Figure 3. While a nuclear staining pattern is observed with the Ki-67 and BD ProEx C biomarkers, the p16^INK4a^ staining pattern is more variable, with staining found in the nucleus, cytoplasm, or both, the significance of which is not fully understood at the present time.

The performance of Ki-67 [16, 42, 67], p16^INK4a^ (reviewed in [24]), and BD ProEx C [62–64, 66, 68–72] in histological applications has been evaluated in many studies. The comparison of biomarker performance in the detection of high-grade cervical disease is difficult due to the variability in specimens, study design, antibodies, and scoring algorithms utilized. A limited number of studies, however, have analyzed the performance of Ki-67, p16^INK4a^, and BD ProEx C immunohistochemistry on the same sample sets, allowing a preliminary comparison to be made, although scoring algorithms are inconsistent [62–66] (Table 2). The performance of the three biomarkers was fairly comparable, with BD ProEx C and p16^INK4a^ tending to have better diagnostic value than Ki-67. Two studies suggest that BD ProEx C has improved efficacy in the detection of low-grade lesions [62, 63]. Of the three studies using histological tissue [62–64], each concludes that the BD ProEx C/p16^INK4a^ biomarker combination appears to have the best overall performance for triaging diagnostically difficult atypias in which the differential diagnosis includes HSIL, and for the detection of clinically relevant disease compared with the single biomarkers or other biomarker combinations.

Negri et al. [68] evaluated the utility of p16^INK4a^, ProEx C, and Ki-67 for the diagnosis of endocervical adenocarcinoma and its precursors. p16^INK4a^ was at least focally expressed in 93% (14/15) of invasive adenocarcinomas, 100% of AIS (29/29), and 32% (7/22) negative samples. ProEx C and Ki-67 both scored positive in all adenocarcinomas (15/15) and AIS (29/29), and in 36% (8/22) and 27% (6/22) of negative samples, respectively. p16 and Ki-67 individually stained positive in 94% (15/16) of glandular dysplasia cases, with 87.5% positivity (14/16) detected with ProEx C. The score differences between neoplastic and nonneoplastic samples were highly significant for each marker (*P* < 0.001), and each biomarker was shown to be useful for the diagnosis of neoplastic lesions of the glandular epithelial of the cervix uteri.

In a study examining cell block preparations [65], BD ProEx C was found to have a higher PPV for high-grade dysplasia/carcinoma (89%) than p16 (50%), with a NPV value of 93% for ProEx C compared with 100% for p16^INK4a^. The study concluded that BD ProEx C had a better overall performance in differentiating NILM versus HSIL/SCC compared with p16^INK4a^ with the added benefit of having clean nuclear staining.

## 5. Evaluation of Progression Risk

One major issue in the management of cervical cancer is the evaluation of progression risk of dysplastic lesions. Patients with CIN1 must be periodically followed due to the risk of progression to high-grade lesions or carcinoma. As 70–80% of low-grade lesions spontaneously regress and not all high-grade lesions progress [73], some women may undergo unnecessary treatment or have a delay in receiving treatment. Thus, the identification of biomarkers to select women truly at risk of lesion progression and in need of treatment could lead to tremendous cost savings and eliminate patient anxiety.

A limited number of studies have reported that the application of Ki-67 immunoquantitative analyses of CIN1 and CIN2 can predict disease progression, with the best features to predict progression being the 90th percentile of the stratification index and the percentage of Ki-67 positive cells in the middle third layer of the epithelium [74, 75].

Recent prospective end point studies have shown that p16^INK4a^ positive low-grade lesions have a higher risk of progression than negative lesions, although this correlation was certainly not absolute [76–79]. Additional prospective data are necessary, however, to confirm this association. Ozaki et al. [80] examined expression of the p16^INK4a^ and ProEx C biomarkers in premalignant lesions to determine which markers could help in prediction of the progression of CIN1. Expression of both markers was significantly higher in the progression group compared to the regression group, being sensitive (86%) and moderately specific (60% and 61%, resp.) in predicting CIN1 progression. Hariri and Hansen [81] compared the prognostic value of p16^INK4a^ to BD ProEx C and HPV ISH in CIN1 cases and found BD ProEx C to be a reliable marker for prediction of 6-year outcome, with an NPV of 95.3% compared with 88.6% for p16 and 87.5% for HPV ISH and a PPV of 51% compared with 40.4% and 67.9%, for p16 and HPV ISH, respectively.

Interestingly, CIN2/3 cervical histology specimens have been described with very strong p16^INK4a^ positivity, but only very focal Ki-67 staining, indicating a presence of a small subset of HSILs with low proliferative activity [82, 83]. Cases like this may represent the first stages of HSIL regression.

HPV L1 is a capsidic protein that is expressed in the early, productive phase of HPV infection, but progressively lost during cervical carcinogenesis. An analysis of thin-layer preparations showed that the L1 capsid protein is produced in about 80% of mild-to-moderate dysplasias, whereas it could only be detected in about 25% of higher-grade dysplasias using immunological methods [28], due, in part, to HPV integration that accompanies the development of cervical neoplasia. The detection of the HPV L1 capsid protein in combination with p16^INK4a^, to confirm the association of the lesion with HPV, has been reported to serve as a prognostic marker that can differentiate between patients who will undergo a transition from a precursor lesion to cancer and those whose lesions will regress [39, 84]. While the data is still preliminary, in cases where the grade of lesion is morphologically difficult to assess, the L1 pattern may be helpful for deciding the appropriate management of women. L1-negative HPV high-risk positive mild and moderate lesions have an extremely low probability to regress spontaneously (5%) in contrast to the L1 positive cases showing a low malignant potential [41].

## 6. Summary

A number of protein biomarkers are currently available to assist in improving the clinical performance of cervical cancer screening. The recent introduction of prophylactic HPV vaccines will eventually reduce the incidence of cervical cancers and its malignant precursors, therefore, increase the importance of biomarkers in future cervical cancer screening programs to identify for treatment only those women truly at high risk for developing cervical cancer. It is anticipated that the use of these biomarkers can be applied both as a reflex test from an atypical Pap specimen but also as a primary screen to improve the overall accuracy of the Pap test. The introduction of HPV primary screening programs will necessitate the use of a reflex test with high specificity to triage the high number of HPV positive tests. It is believed that biomarkers will also serve an important role in the optimization of this alternative screening algorithm. Current translational research investigations are continuing to discover, characterize, and validate such biomarkers for these anticipated applications.

## Figures and Tables

**Table 1:** Biomarkers used in cervical cancer screening and diagnosis.

Biomarker	Staining	Cellular process detected	Reported use of biomarker
	*Pattern*		
Ki-67	Nuclear	Increased Ki-67 staining reflects increased epithelial cell proliferation found in HPV-infected tissues.	(i) Measure of cell proliferative capacity. (ii) Recognizes tissues involved by HPV and extent of Ki-67 immunostaining generally parallels increasing grades of dysplasia. (iii) Predominantly used in histology applications.

p16^INK4a^	Nuclear and cytoplasmic	p16 levels increased in response to irregular cell cycle inactivation resulting from the disruption of interaction of pRb with transcription factor E2F in the presence by the HPV E7 oncogene.	(i) Detection can serve as a surrogate biomarker for persistent infection with high-risk HPV. (ii) Triage of equivocal cytology findings can facilitate identification of abnormal cells in cytology preparations. (iii) Aid in interpretation of histological material. Limited evidence for use as a predictor of disease progression in histology specimens.

BD ProEx C	Nuclear	Increased cellular levels of MCM2 and TOP2A due to aberrant transcription of S-phase proteins resulting from the interaction of HPV E6 and E7 oncoproteins with cell cycle proteins p53 and Rb.	(i) Marker of cells with proliferative capacity. (ii) Triage from abnormal cytology to increase PPV over cytology alone or HPV triage for detection of CIN2+ disease. Can also facilitate identification of abnormal cells in cytology preparations. (iii) Use in histology to distinguish true dysplasia from mimics such as reactive/reparative changes, immature squamous metaplasia, and atrophy.

Cytoactiv HPV L1	Nuclear	HPV L1 capsid protein found in mild-to-moderate dysplasias, but lost in higher-grade intraepithelial neoplasias.	(i) Possible prognostic marker to identify early dysplastic lesions most likely to progress to high-grade disease.

**Table 2:** Biomarker positivity by histological grade of cervical intraepithelial lesion.

*Study*	*Biomarker*	*WNL*	*CIN1*	*CIN2+*
	Ki-67	2/14 (14%)	29/34 (85%)	14/14 (100%)
Shi 2007 et al.[62]	p16^INK4a^	0/14 (0%)	26/34 (77%)	14/14 (100%)
	BD ProEx C	0/14 (0%)	32/34 (94%)	11/14 (79%)
	BD ProEx C/p16	0/14 (0%)	34/34 (100%)	14/14 (100%)

	Ki-67	0/37 (0%)	6/22 (27%)	34/37 (91%)
Badr 2008 et al. [63]	p16^INK4a^	2/38 (5%)*	8/23 (35%)**	35/37 (93%)
	BD ProEx C	1/38 (3%)	11/23 (48%)	34/37 (92%)
	BD ProEx C/p16	3/35 (9%)	15/23 (65%)	37/37 (100%)

	Ki-67	11/23 (48%)	ND	34/36 (94%)
Pinto 2008 et al. [64]	p16^INK4a^	13/35 (37%)	ND	51/61 (84%)
	BD ProEx C	10/35 (29%)	ND	52/60 (87%)
	BD ProEx C/p16	9/23 (39%)	ND	33/36 (92%)

	Ki-67	14/29 (48%)^a^	22/27 (82%)	15/16 (94%)
Halloush 2008 et al. [65]	p16^INK4a^	19/29 (66%)^b^	25/27 (92%)	19/19 (100%)
	BD ProEx C	2/29 (7%)^c^	2/27 (7%)	16/19 (84%)

	Ki-67	6/26 (23%)	10/21 (48%)	76/85 (89%)
Conesa-Zamora 2009 et al. [66]	p16^INK4a^	4/28 (14%)	12/19 (63%)	74/85 (87%)
	BD ProEx C	3/25 (12%)	10/19 (53%)	70/80 (88%)

WNL: within normal limits; CIN: cervical intraepitelial neoplasia; ND: not determined; *Spotty staining; **5/23 spotty staining; ^a^12/29 <1% staining, 2/29 ≥1% staining; ^b^19/29 <10% staining; ^c^1/29 <10%.
